# Alcohol Use Disorder and Co-Occurring Mental Health Conditions

**DOI:** 10.35946/arcr.v40.1.08

**Published:** 2019-12-31

**Authors:** Laura E. Kwako, Jenica Patterson, Ihsan M. Salloum, Ryan S. Trim

**Affiliations:** Laura E. Kwako, Ph.D., is a program director in the Division of Treatment and Recovery Research, National Institute on Alcohol Abuse and Alcoholism, Rockville, Maryland; Jenica Patterson, Ph.D., is a program officer in the Division of Neuroscience and Behavior, National Institute on Alcohol Abuse and Alcoholism, Rockville, Maryland; Ihsan M. Salloum, M.D., is a professor in the Department of Psychiatry and Behavioral Sciences, chief of the Division of Substance and Alcohol Abuse, and director of the Addiction Psychiatry Fellowship Program, University of Miami Miller School of Medicine, Miami, Florida; Ryan S. Trim, Ph.D., is an associate professor in the Department of Psychiatry, University of California San Diego School of Medicine, La Jolla, California

**Figure:**
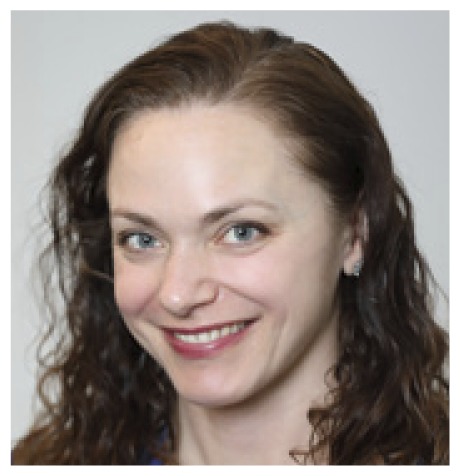
Laura E. Kwako

**Figure:**
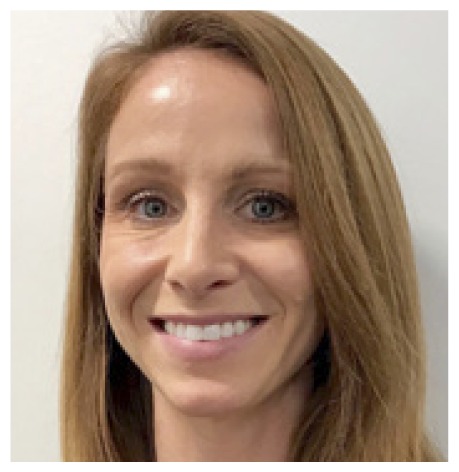
Jenica Patterson

**Figure:**
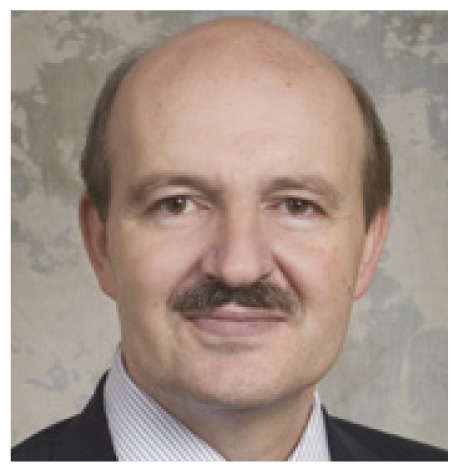
Ihsan M. Salloum

**Figure:**
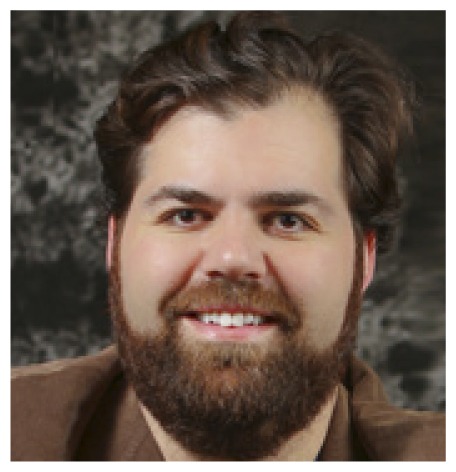
Ryan S. Trim

This issue of *Alcohol Research: Current Reviews* (ARCR) delves into studies on co-occurring alcohol use disorder (AUD) and mental health conditions, exploring how this co-occurrence affects symptom severity, prognosis, and outcomes. Increased risk because of co-occurrence, challenges because of disorder heterogeneity, and efficacy of treatment interventions are reviewed.

Among people with AUD, depressive disorders are one of the most common co-occurring psychiatric conditions. In **Alcohol Use Disorder and Depressive Disorders**, McHugh and Weiss discuss the prevalence, course, and treatment of co-occurring AUD and depressive disorders. They also examine disproportionately affected populations, developmental pathways to co-occurrence, and the challenges of diagnosis because of overlapping symptoms.

In the “Focus On” review **Suicidal Behavior: Links Between Alcohol Use Disorder and Acute Use of Alcohol**, Conner and Bagge explore the connection between alcohol use and suicidal behavior. Postmortem investigations on individuals who have died by suicide have found that AUD is prevalent among this group and that acute use of alcohol was often present. In their review, Conner and Bagge discuss the role alcohol plays in increasing risk for suicidal behavior and consider the efficacy of various interventions.

Anker and Kushner consider the association between AUD and anxiety in **Co-Occurring Alcohol Use Disorder and Anxiety: Bridging Psychiatric, Psychological, and Neurobiological Perspectives.** They review the research on the psychiatric classifications of alcohol misuse and negative affect and examine the relationship between negative affect and alcohol use from a neurobiological standpoint.

Weera and Gilpin, in the “Focus On” review **Biobehavioral Interactions Between Stress and Alcohol**, examine how brain stress systems mediate the effects of stress on alcohol drinking. They summarize key findings from animal models and suggest that brain stress systems may be useful targets for medications development.

In **Alcohol Use Disorder and Antisocial and Borderline Personality Disorders**, Helle and colleagues focus on co-occurring AUD and personality disorders. They discuss prevalence rates, potential explanations and causal models of comorbidity, and the status of treatment research. Helle and colleagues also discuss how personality traits, symptoms, and etiology can affect diagnosis and treatment.

In **Alcohol Use Disorder and Schizophrenia or Schizoaffective Disorder**, Archibald and colleagues explore schizophrenia spectrum disorders and their high co-occurrence with AUD. They describe how shared neurobiological mechanisms may explain the co-occurrence of these disorders. These authors suggest that combining pharmacologic interventions with other therapeutic modalities may address both issues more effectively.

Yule and Kelly, in **Integrating Treatment for Co-Occurring Mental Health Conditions**, consider the prevalence and treatment of co-occurring AUD and mental health conditions. They discuss screening tools, assessment, and the development of different treatment approaches. They also review the challenges to effective treatment and emphasize the importance of treating of both conditions.

## From the Editor in Chief

After much consideration, NIAAA leadership and journal staff have made the decision that ARCR will transition to an online-only publication format in 2020. An analysis of the readership found that although print subscriptions have declined in recent years, readers regularly access ARCR content online through PubMed, PubMed Central, and the ARCR website. The online-only format will allow for more frequent and timely publications, permit reviews of emerging areas of alcohol research, and reduce ARCR’s carbon footprint. As an open-access journal, ARCR will continue to be freely available to the public and the alcohol research community.

—Troy J. Zarcone, Ph.D.

